# Efficacy of a Computer-Assisted Cognitive Rehabilitation Intervention in Relapsing-Remitting Multiple Sclerosis Patients: A Multicenter Randomized Controlled Trial

**DOI:** 10.1155/2017/5919841

**Published:** 2017-12-31

**Authors:** Lambros Messinis, Grigorios Nasios, Mary H. Kosmidis, Petros Zampakis, Sonia Malefaki, Katerina Ntoskou, Anastasia Nousia, Christos Bakirtzis, Nikolaos Grigoriadis, Philippos Gourzis, Panagiotis Papathanasopoulos

**Affiliations:** ^1^Neuropsychology Section, Department of Neurology, University of Patras Medical School, 26504 Patras, Greece; ^2^Department of Psychiatry, University Hospital of Patras and University of Patras Medical School, 26504 Patras, Greece; ^3^Department of Speech and Language Therapy, Higher Educational Institute of Epirus, Ioannina, Ioannina, Greece; ^4^Lab of Cognitive Neuroscience, School of Psychology, Aristotle University of Thessaloniki, Thessaloniki, Greece; ^5^Department of Radiology, University of Patras Medical School, 26504 Patras, Greece; ^6^Department of Mechanical Engineering & Aeronautics, University of Patras, 26504 Patras, Greece; ^7^Rehabilitation Unit for Patients with Spinal Cord Injury, “Demetrios and Vera Sfikas”, Department of Medicine, University of Patras, 26504 Patras, Greece; ^8^B'Department of Neurology and the MS Center, AHEPA University Hospital of Thessaloniki, Thessaloniki, Greece; ^9^University of Patras Medical School, 26504 Patras, Greece

## Abstract

Cognitive impairment is frequently encountered in multiple sclerosis (MS) affecting between 40–65% of individuals, irrespective of disease duration and severity of physical disability. In the present multicenter randomized controlled trial, fifty-eight clinically stable RRMS patients with mild to moderate cognitive impairment and relatively low disability status were randomized to receive either computer-assisted (RehaCom) functional cognitive training with an emphasis on episodic memory, information processing speed/attention, and executive functions for 10 weeks (IG; *n* = 32) or standard clinical care (CG; *n* = 26). Outcome measures included a flexible comprehensive neuropsychological battery of tests sensitive to MS patient deficits and feedback regarding personal benefit gained from the intervention on four verbal questions. Only the IG group showed significant improvements in verbal and visuospatial episodic memory, processing speed/attention, and executive functioning from pre - to postassessment. Moreover, the improvement obtained on attention was retained over 6 months providing evidence on the long-term benefits of this intervention. Group by time interactions revealed significant improvements in composite cognitive domain scores in the IG relative to the demographically and clinically matched CG for verbal episodic memory, processing speed, verbal fluency, and attention. Treated patients rated the intervention positively and were more confident about their cognitive abilities following treatment.

## 1. Introduction

Cognitive impairment is frequently encountered in multiple sclerosis (MS) affecting between 40–65% of individuals, irrespective of disease duration, severity of physical disability, and at both the earlier and later disease stages [1, 2]. Moreover, cognitive dysfunction in this population may have a significant negative impact on quality of life [3], activities of daily living and independence [4], and employment status [5]. Furthermore, past and current pharmacological treatments have shown inconsistent findings in alleviating cognitive impairment in individuals with MS requiring further clarification [6]. This inconsistency regarding the effects of pharmacological interventions on cognition, coupled with the reduced ability to effectively handle everyday tasks, loss of employment and social interaction capacity, and overall poorer quality of life prioritizes the need for utilizing potentially more effective nonpharmacological, neurobehavioural interventions to address cognitive dysfunction and everyday functioning abilities.

Neurobehavioral interventions utilizing cognitive rehabilitation have shown favorable effects on MS patients' cognitive performance and other related skills and, in some cases, have managed to generalize these positive effects to an MS individual's everyday life functioning ability [7–10]. While as described previously, there is evidence to support cognitive rehabilitation interventions in the MS population, the results of past and present clinical trials have been marred by numerous methodological limitations. These include lack of appropriate control groups and objective neuropsychological status assessment at baseline, utilization of inappropriate randomization methods, single-site studies, and inconsistency regarding the specific target of the rehabilitation intervention and outcome measures (especially as regards the use of ecologically valid interventions and measures) [11]. Therefore, it becomes obvious that there is a need for rigorous new cognitive rehabilitation studies that may overcome some of these limitations and provide robust evidence regarding the efficiency of such interventions.

The purpose of this study was to investigate the effectiveness of a 10 week (2 days a week for approximately 60 minutes) computer-assisted cognitive rehabilitation intervention, utilizing the RehaCom® software (RehaCom Cognitive Therapy Software. https://www.rehacom.co.uk) on cognitive functioning in Greek relapsing-remitting MS (RRMS) patients, who on baseline assessment had mild to moderate cognitive impairments. We hypothesized that patients (IG) receiving the individualized 10-week intervention will show improved pre- to postintervention performance on neuropsychological measures in the related trained cognitive domains relative to control group (CG) participants who will receive only usual-standard clinical care. Moreover, we hypothesized that the positive training effects on specific cognitive domains (episodic memory, information processing speed/attention and executive functions) would be retained over time (6 months in this case) providing evidence on the long-term benefits of such interventions. We also hypothesized that control participants will show either further cognitive decline or remain cognitively stable as the period of the intervention may be inadequate to produce significant cognitive changes in these patients.

## 2. Methods

### 2.1. Participants

Between March of 2014 and December of 2015, 98 patients who had been previously diagnosed with relapsing-remitting MS (RRMS) based on the McDonald criteria [12], attending either the outpatient neurology department at the University Hospital of Patras in Greece or the “Society of friends of patients with multiple sclerosis” situated in Ioannina, and who reported cognitive difficulties or were judged by clinical neurological evaluation to have cognitive deficits were referred for neuropsychological assessment at the outpatient memory and neuropsychological unit of the same hospital or the laboratory of audiology, neurotology, and neurosciences of the Higher Educational Institute of Epirus, Ioannina, Department of Speech and Language Therapy. Clinicians assessing patients at both sites were supervised by the clinical neuropsychologist (LM) and lead consulting neurologists (PP) in Patras and (GN) in Ioannina.

Of the 98 patients initially screened, fifty-eight were included in the study after meeting specific inclusion criteria. These patients were randomly assigned to either receive treatment with the RehaCom software (IG; *n* = 32) or placed in the control group condition (CG; *n* = 26) and received usual-standard clinical care. Demographic and clinical characteristics of both groups at baseline are provided in Table 1.

All patients met the criteria for the diagnosis of MS according to [12]. Additional study inclusion criteria were (i) patients aged between 21 and 60, (ii) educational level of at least 6 years (primary school graduates in Greece), (iii) relapsing-remitting MS (RRMS), (iv) EDSS score of between 0–5, (v) cognitive deficit on at least one domain of the Central Nervous System Vital Sign neuropsychological screening battery [13], (vi) native Greek speakers, (vii) provision of written informed consent to take part in the study, and (viii) IQ score of ≥80 on the Greek-validated Wechsler Abbreviated Scale of Intelligence (WASI) [14]. Exclusion criteria were as follows: (i) ongoing major psychiatric disorders (e.g., psychotic symptoms or disorders, illegal drugs, or alcohol abuse); (ii) presence of another neurological disorder (e.g., dementia, stroke, epilepsy, and traumatic brain injury resulting in a loss of consciousness for more than 30 minutes); (iii) Mini-Mental State Examination score MMSE ≥ 24; (iv) one or more exacerbations in the 3 months prior to enrollment and immunological or immunosuppressant treatment initiated within 4 months prior to enrollment or treated with cognitive rehabilitation in the 12 months prior to enrollment; (v) initiation of psychotropic medications or medications for spasticity, tremor, bladder disturbances, and fatigue, if already taking such medications, doses and schedules had to be held constant during the study period; and (vi) normal or corrected hearing and vision.

### 2.2. Procedure

After been initially evaluated on a brief screening neuropsychological battery (Central Nervous System Vital Signs—CNSVS [13, 15]), patients with a diagnosis of RRMS that were found to have cognitive deficits on at least one domain of the CNSVS (performance between the 2nd and 8th percentile based on CNSVS demographically corrected normative data) were informed of the opportunity to participate in a 10-week cognitive rehabilitation intervention by the lead consulting neurologists (PP) and (GN) or clinical neuropsychologist (LM) supervising the study and were invited to take part after providing written informed consent.

**Figure 1:**
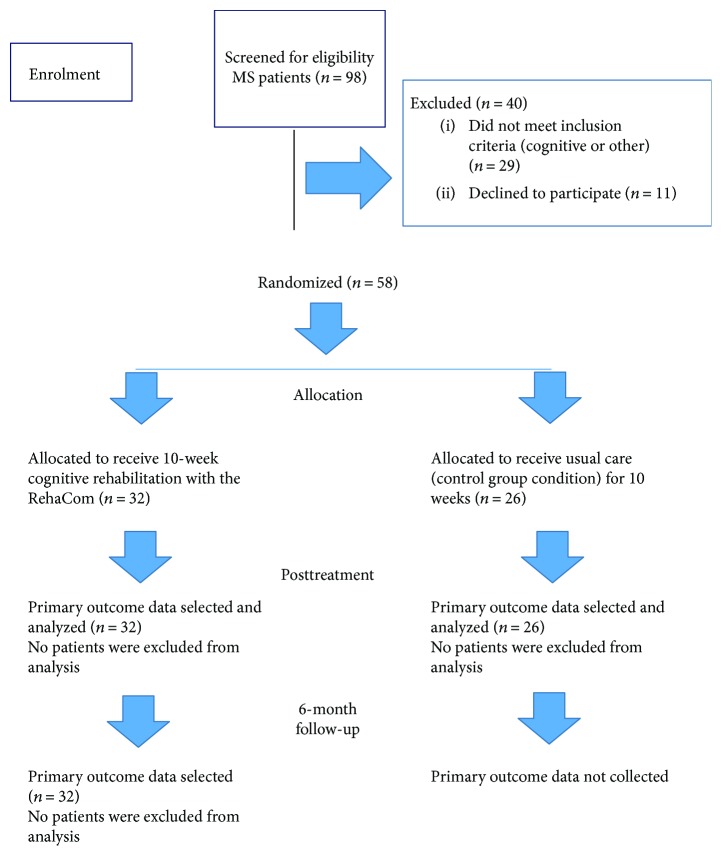
Consort flow diagram.

In order to overcome the limitations of recruiting patients from only one site, Southwestern Greece in this particular case, and to provide a more representative sample of MS patients, RRMS patients included in the intervention protocol, as mentioned previously, were also recruited from a second site, the national Society of MS attendees in Northwestern Greece known as the “Society of friends of patients with multiple sclerosis” situated in Ioannina, by following the exact same protocol as the patients recruited from Southwestern Greece. Eligible patients were randomized by a computer -generated, site-stratified, independent randomization schedule to either undergo cognitive rehabilitation (IG; intervention group) with the RehaCom software or were placed in the placebo arm (CG; control group) and spent the same portion of time (10 weeks) receiving usual clinical care. Before initiating the intervention (pretreatment), patients in both groups were administered a flexible battery of neuropsychological tests and measures of mood. Both groups were then evaluated within one week after completing the intervention (posttreatment), and the RehaCom-treated group was also evaluated at a six month follow-up (see Figure 1). All patients were noncompensated volunteers.

In both settings, qualified clinicians which had previously attended training sessions in order to ensure uniform test administration and application of the rehabilitation intervention, following a strict protocol, and under the supervision of an experienced clinical neuropsychologist (LM) administered the screening CNSVS battery and the flexible comprehensive neuropsychological battery of tests with well-validated psychometric properties in MS individuals and all other measures (excluding the EDSS scale which measures disability and was administered by specialist neurologists) at all the evaluation stages. Moreover, they conducted the rehabilitation interventions for the entire 10-week duration. The participants and clinicians taking part in the assessments and intervention were not blind to the allocated treatments. However, scoring of neuropsychological measures at baseline, posttreatment, and at 6-month follow-up was performed by two blinded observers, in order to avoid interrater variability.

Thirty-two participants diagnosed with RRMS completed the intervention, whereas twenty-six were included in the control group and received usual-standard clinical care for the entire 10 weeks as mentioned previously. Six months following the intervention and continuing with usual clinical care for this time period, only patients that had undergone cognitive rehabilitation were evaluated neuropsychologically in a follow-up session in order to establish the effects of the intervention over time. Furthermore, although no formal posttreatment or follow-up questionnaire was used as an outcome measure to determine the personal benefit of each patient gained from the intervention, we informally asked treated patients to provide feedback regarding the intervention on four verbal questions at posttreatment assessment. The questions were (i) how much have you personally benefited from this type of treatment? (ii) have your cognitive difficulties improved after the program? (iii) has this program helped you to improve your everyday life activities (e.g., can you now remember more items of a shopping list without writing down the list or do you now need less time to complete mental tasks or plan a trip)? and (iv) would you recommend this intervention to other MS patients? Patients had to rate their response on a Likert-type Scale ranging from 1–5, where 1 was indicative of no benefit, 2 (minor benefit), 3 (medium benefit), 4 (moderate benefit), and 5 (large benefit). Patients assigned to the control condition were for ethical reasons provided the opportunity to participate in a cognitive rehabilitation intervention similar to the one utilized in this study once they completed the research protocol. The research protocol was approved by the ethics committee of the University of Patras Medical School and was conducted in accordance with the principles of the Declaration of Helsinki (WMA, 2013). Patients recruited from both sites provided written informed consent to take part in the study.

### 2.3. Instruments: Outcome Assessment

#### 2.3.1. Clinical Assessment

Clinical characteristics of MS patients were assessed by specialist neurologists with significant experience in the MS population. These neurologists provided the diagnosis of MS based on the [12] criteria, type of disease course, disability rating on the EDSS scale [15], fatigue rating on the Greek-validated Fatigue Severity Scale (FSS) [16], types of medications patients were taking, duration of illness, and differential diagnostic issues and also screened patients to ensure eligibility of inclusion and exclusion criteria (with the exception of cognitive criteria and mood). If deemed necessary, patients were also assessed psychiatrically to ensure correct differential diagnosis of behavioral and mood disorders and to exclude patients with ongoing major psychiatric disorders.

#### 2.3.2. Initial Screening Assessment of Cognitive Functions and Intelligence Level

As noted previously, all MS patients that were referred for neuropsychological assessment were initially screened on a brief neuropsychological battery (Central Nervous System Vital Signs—CNSVS [12]) in order to evaluate their cognitive status and to determine whether they had impaired cognitive performance (one of the study inclusion criteria) on any of the CNSVS-tested domains defined as performance between the 2nd and 8th percentile based on demographically corrected normative data. The CNSVS battery provides core neuropsychological assessment utilizing seven neuropsychological tests [12]. These include the Verbal and Visual Memory Test, Finger Tapping Test, Symbol Digit Coding Test, Stroop Test, Shifting Attention Test, and Continuous Performance Test.

Intelligence level of MS patients at this stage was also estimated by administering the vocabulary and matrix reasoning subscales of the Greek-adapted version of the Wechsler Abbreviated Scale of intelligence WASI [14, 17]. The vocabulary subscale is a good measure of crystallized intelligence, correlates well with general intellectual ability, and is relatively insensitive to cortical insults (i.e., considered a good measure of premorbid intellectual ability). For this reason, the demographically corrected *T*-score of the vocabulary scale was used as an estimate of premorbid intelligence level in this study. The Matrix Reasoning subscale is a measure of nonverbal fluid reasoning and correlates well with general intellectual ability. These two subscales yield an estimated full-scale IQ.

At this screening stage, patients were further administered the Greek-validated version of the Mini-Mental State Examination (MMSE) [18]. The MMSE assesses a restricted set of cognitive functions simply and quickly and is utilized as a dementia-screening measure in everyday clinical practice. Recently, Solias et al. [19] provided MMSE “cutoff scores” for discriminating demented patients in Greece based on age- and education-corrected norms. An MMSE score of ≥24 was one of the stipulated study inclusion criteria.

#### 2.3.3. Neuropsychological Assessment

Both groups of patients were administered a comprehensive flexible battery of neuropsychological tests at baseline and within one week of completing the RehaCom treatment phase. The RehaCom-treated group was also assessed 6 months following the completion of the rehabilitation intervention after receiving only the usual clinical care for this period. The main criterion for selecting the cognitive measures to be utilized in this study were their use specifically for this population in routine clinical care and for research purposes, see for example [20–23]. Moreover, the selected cognitive measures assess domains that are normally impaired in MS individuals, independent of disease duration and disability status. This included tests of attention, mental processing speed, verbal fluency/language, verbal and visuospatial memory, and executive functions. All neuropsychological tests were administered using standard procedures in single sessions. To minimize retest effects, alternative forms of the tests were used when available.  Table 2 provides a summary of the utilized neuropsychological test battery arranged by cognitive function/domain assessed.

#### 2.3.4. Assessment of Mood

The Beck Depression Inventory-Fast Screen for Medical Patients (BDI-Fast Screen) [31, 32] was administered in order to assess the severity of depression. The BDI-Fast Screen is a 7-item self-report case-finding instrument that screens for severity of depression that corresponds to the psychological or nonsomatic criteria for diagnosing major depression disorders as listed in the DSM-IV [33] in adults and adolescents. It consists of seven items extracted from the 21-item Beck Depression Inventory-II [34]. The administration procedure used was the one suggested by Beck et al. and Strauss et al. [31, 35], using a Greek-translated and adapted version [32], with Cronbach's internal reliability coefficient (*a* = 0.82). The dependent variable in this study included the sum of the highest ratings for each of the seven items (maximum score = 21). The BDI-Fast Screen has been validated in multiple sclerosis patients [36]. More specifically, it discriminated individuals with MS that were receiving treatment for depression from untreated MS patients with neurological symptoms. Moreover, the authors with their findings support its concurrent and discriminative validity in the MS population [36].

#### 2.3.5. Assessment of Fatigue

Fatigue was assessed with the Fatigue Severity Scale (FSS), a 9-item self-assessment scale [37]. The scale was recently adapted and validated in Greek MS patients and found to be reliable and valid for this population [16]. Respondents indicate the fatigue level they experienced throughout the last two weeks. The questions are related to how fatigue interferes with certain activities and rates its severity. The items are scored on a 7-point scale with 1 = strongly disagree and 7 = strongly agree. The scoring is done by calculating the average response to the questions (adding up all the answers and dividing by nine). The minimum score = 1 and maximum score possible = 7. A higher score is indicative of greater fatigue severity. In a recent validation study by Learmonth et al. [38], a mean FSS score ≥ 4 was indicative of substantial fatigue in 77% of the MS patients.

#### 2.3.6. Treatment Intervention

As noted previously, MS patients that were eligible to take part in the study were randomized to either receive specific-computerized cognitive remediation training-cognitive rehabilitation (*n* = 32), over a period of 10 weeks, with 2 weekly 60-minute sessions on an individual basis or usual clinical care standard treatment (*n* = 26) for the same time period. The study is a multicentric (2 centers), randomized controlled trial investigating the efficacy of cognitive functional training in RRMS patients. This approach aims to improve cognitive functioning by restoring or improving network efficiency in the brain

*(1) Treatment Intervention: Computer-Assisted Cognitive Rehabilitation (RehaCom modules)*. The treatment consisted of 20 individualized one-hour sessions over a 10-week period, with a frequency of two sessions per week. The rehabilitation program was conducted by trained clinicians, either speech and language therapists or psychologists, and supervised by a clinical neuropsychologist (LM), on a desktop computer with a large screen. The computer was connected with a special input panel using the commercially available RehaCom software package (RehaCom Cognitive Therapy Software. https://www.rehacom.co.uk), which has been utilized extensively in Europe over the last couple of years for the purpose of providing computer-assisted cognitive rehabilitation. The panel keyboard that is utilized limits the interference of motor and coordination impairments. Moreover, the software which has over 20 modules is available in many languages, including Greek. In Greece, the software is available commercially at Ostracon. For more details about this product see the Ostracon website at http://ostraconmed.com/ostracon-proionta/gnostiki-apokatastasi/rehacom/gia-ton epaggelmatia/.

It provides the opportunity to train patients on several levels of difficulty and length of sessions, and according to whether the patient succeeds or fails the task, the difficulty levels are automatically adjusted to meet the patient's needs. Once the training is completed, the therapist can review the session from the results screen. The data can be presented in a variety of ways including charts, graphs, and comparisons. The most common format results are level of progression, number of mistakes, and time utilized for each cognitive task. By analyzing the data thoroughly, the therapist is able to identify particular weaknesses of the patient and address this further in the training. For this specific study, as most of our MS patients that took part in the intervention were impaired in more than one cognitive domain but mostly on episodic memory, information processing speed/attention, and executive functions, the intervention was balanced over the 10-week period in order to train all domains equally. A detailed description of the RehaCom modules used in the cognitive rehabilitation intervention is provided in the Appendix.

*(2) Control Group: Standard Clinical Care*. MS patients that were randomized to receive standard or usual clinical care continued taking their prescribed medication and all other related treatments (e.g. physiotherapy, psychotherapy), and all other clinical or referral services were available to them as usual for the entire 10 weeks that the intervention group received cognitive training. As in the University Hospital of Patras or the laboratory of audiology, neurotology, and neurosciences of the Higher Educational Institute of Epirus, specific interventions for cognitive difficulties in MS patients are not offered on a standard basis, these patients did not receive any specific cognitive rehabilitation for their cognitive problems. This group of patients for ethical reasons was offered the opportunity to undertake cognitive rehabilitation after completion of the study period.

### 2.4. Statistical Analysis

We initially computed the basic descriptive statistics and the 95% confidence intervals of the demographic (age, education level, gender, and Wechsler Abbreviated Scale of intelligence: full 2 scale IQ and Vocabulary subscale *T*-score), clinical (Expanded Disability Status Scale, Mini-Mental State Examination, Beck Depression Inventory-Fast Screen, duration of illness, medication regimen at enrolment, and Fatigue Severity Scale), and neuropsychological variables (Trail Making Test parts A and B, Selective Reminding Test, Brief Visuospatial Memory Test-Revised, Symbol Digit Modalities Test, Greek Verbal Fluency Test (semantic and phonemic), and Stroop Neuropsychological Screening Test-colour word task). Next, the normality assumption of the data was tested using the Shapiro-Wilk test since it is more powerful than the most commonly used in practice Kolmogorov-Smirnov test [39]. When the hypothesis of normality was rejected, the nonparametric Mann–Whitney *U* test was used to examine the differences between our two groups (intervention and control group); otherwise, the standard independent sample *t*-test was used. For the comparison of dependent populations, the nonparametric Friedman test was used whenever the normality assumptions were rejected and the paired samples *t*-test in all other cases. The Pearson correlation coefficient was used in order to measure correlations between neuropsychological and disease variables, depression, and fatigue. Furthermore, due to the use of multiple cognitive measures to assess cognitive functions, we decided to calculate composite scores and formulate composite variables (cognitive domains) for verbal episodic memory, attention, verbal fluency, and processing speed, by transforming raw neuropsychological test scores obtained from the neuropsychological assessment to form composite domain *z*-scores. In order to extract the new composite variables, the internal consistency of these variables was measured using Cronbach's alpha. As the internal consistency of all extracted composite domains was considered acceptable (*α* > 0.60), the new variables were derived as a weighted sum of the *z*-scores of the initial neuropsychological variables. We also applied a mixed effect ANOVA in order to compare the mean cognitive domain performance difference between the intervention and control group (between subject's factor) and the time points (baseline and posttreatment) that patients were cognitively evaluated (within subject's factor). Moreover, the interaction of these two factors was evaluated by a two-way mixed ANOVA. Statistical analyses were conducted using the statistical package SPSS 22.0 for Windows.

## 3. Results

### 3.1. Comparison of Demographic and Clinical Characteristics at Baseline (Pretreatment)

In general, there was a higher proportion of females compared to males that took part in the study. The percentage of females was higher in both groups (68.75% for the rehabilitation and 69.23% for the control group), something that was expected due to the higher female to male ratio in the MS population in general. However, the proportion/ratio of females between the two groups was not significantly different, [*x*^2^(1) = 0.002, *p* = 0.969]. We then investigated the normality distribution of our data with the *Shapiro-Wilk normality test*. For the variables age and BDI-FS (depression level), the null hypothesis could not be rejected; therefore, we used the parametric independent samples *t*-test to test group differences on this variable. In contrast for the variable level of education, WASI (full IQ 2 scale; intelligence level), WASI vocabulary scale *T*-score (estimated premorbid intelligence level), FSS (fatigue severity), EDSS (disability level), MMSE, and duration of illness, we rejected the null hypothesis and used the nonparametric Mann–Whitney *U* test to compare these variables. We did not find significant differences between the two groups on baseline (pretreatment) assessment for the variables age [*t*(56) = 379, *p* = 0.706], educational level [*z* = −0.945, *p* = 0.345], intelligence level (WASI 2 scale full IQ) [*z* = −0.959, *p* = 0.338], estimated premorbid intelligence (WASI vocabulary scale) [*z* = −0.959, *p* = 0.338], depression level (BDI-FS) [*t*(56) = 0.179, *p* = 0.859], fatigue severity level (FSS) [*z* = −0.697, *p* = 0.486], [*z* = −0.959, *p* = 0.338], disability level (EDSS) [*z* = −0.126, *p* = 0.899], general cognitive status [*z* = −0.1578, *p* = 0.115], and duration of illness [*z* = −0.1515, *p* = 0.130] (see Table 1 for a detailed description of baseline demographic and clinical characteristics). From the above analysis, we conclude that our two groups were well matched on baseline demographic variables and premorbid intelligence level that may significantly influence outcome measures posttreatment. They also did not differ on important disease-related variables such as duration and course (all had a relapsing-remitting course), neurological disability (EDDS scale), depression (BDI-FS), and fatigue (FSS) severity that have also been reported to negatively impact cognitive performance in MS patients.

### 3.2. Comparison of Neuropsychological Test Scores at Baseline (Pretreatment)

We did not find significant differences between the two groups on baseline (pretreatment) assessment for the variables SRTLR [*t*(56) = 0.201, *p* = 0.842], TMT-B [*t*(56) = 0.201, *p* = 0.604], VFT (semantic) [*z* = −478, *p* = 0.633] and phonemic [*z* = −0.335, *p* = 0.520], SDMT [*z* = −0.916, *p* = 0.360], and BVMT-R [*z* = −0.989, *p* = 0.578]. On the contrary, patients randomized to the intervention group verbally recalled significantly less words (*M*_intervention group_ = 6.09 words versus *M*_control group_ = 7.15 words) after a 20-minute delay period, SRT delay score [*z* = −2.289, *p* = 0.022], and required significantly longer duration (*M*_intervention group_ = 73.50 seconds versus *M*_control group_ = 69.27 seconds) to correctly complete the Trails A test [*z* = −2.294, *p* = 0.020], relative to the control group. These findings imply that the intervention group was marginally more cognitively impaired at baseline assessment (see Table 3 for raw cognitive test performance scores of both groups at baseline, posttreatment, and at 6-month follow-up).

### 3.3. Comparison of Neuropsychological Test Performance for the RehaCom MS-Treated Group between Baseline, Posttreatment, and at 6-Month Follow-Up

We found significant time effects for most of our variables from baseline to posttreatment. Post hoc pairwise comparisons showed that the patients who received functional cognitive training had improved cognitive performance between baseline and posttest on the SRTLR (verbal memory, long-term storage) (*p* = 0.000; with a large effect size; *r* = 0.539), SDMT (processing speed, working memory) (*p* = 0.000; with a large effect size; *r* = 0.522), SRTLR (verbal memory, delay recall) (*p* = 0.000; with a medium effect size; *r* = 0.481), BVMT-R (visuospatial memory, total recall) (*p* = 0.000; with a medium effect size; *r* = 0.469), VFT (semantic) (*p* = 0.003; with a medium effect size; *r* = 0.417), TMT-Α (attention, processing speed) (*p* = 0.000; with a large effect size; *r* = 0.573), TMT-B (executive function, set shifting) (*p* = 0.000; with a large effect size; *r* = 0.506), and SNST-colour word task (executive function, response inhibition) (*p* = 0.000; with a medium effect size; *r* = 0.460). In contrast to the positive time effects of the intervention shown for most of our variables, cognitive training did not significantly improve phonemic fluency even though patients improved their mean phonemic production rate from *M*_baseline_ = 31.88 words versus *M*_posttreatment_ = 33.13 words.

In order to establish whether the treated patients differed in terms of their baseline versus the 6-month follow-up performance, we compared their cognitive measure scores at these time points. The results revealed that treated patients differed significantly on the SRTLTS (*p* = 0.000; with a medium effect size; *r* = 0.469), SRTDR (*p* = 0.001; with a medium effect size; *r* = 0.454), BVMT-R (*p* = 0.001; with a medium effect size; *r* = 0.436), TMT-A (*p* = 0.000; with a large effect size; *r* = 0.509), TMT-B (*p* = 0.000; with a medium effect size; *r* = 0.475), and SNST (*p* = 0.000; with a medium effect size; *r* = 0.448). On three of our outcome variables, nonsignificant differences were established between baseline and follow-up performance on the VFT (semantic) (*p* = 0.424), phonemic (ns), and SDMT (*p* = 0.222). Although patients improved their mean semantic fluency production rate from *M*_baseline_ = 41.03 words versus *M*_follow-up_ = 42.06 words and their mean digit symbol substitution rate in 90 seconds from *M*_baseline_ = 36.91 correct substitutions versus *M*_follow-up_ = 37.50 correct substitutions, this was insufficient to produce statistically significant changes. To examine the long-term effect of the intervention over time, we compared cognitive outcome performance between posttreatment and 6 months' follow-up. Our findings showed that for most of our variables, there were nonsignificant differences between the positive cognitive gains found on posttreatment and follow-up. In contrast, the mean semantic fluency production rate was reduced from *M*_posttreatment_ = 43.56 words versus *M*_follow-up_ = 42.06 words and mean digit symbol substitution rate in 90 seconds from *M*_posttreatment_ = 40.03 correct substitutions versus *M*_follow-up_ = 37.50 correct substitutions, producing statistically significant changes over this time period (see Table 4).

### 3.4. Comparison of Neuropsychological Test Performance for the MS Standard Care Control Group between Baseline and Posttreatment

Our results revealed that in the majority of measures there we no significant changes between pre- and postassessments. An exception was the performance on the mean phonemic fluency production rate that increased from *M*_baseline_ = 29.81 words versus *M*_posttreatment_ = 29.95 words [*z* = −2.365, *p* = 0.018], the mean semantic fluency production rate that decreased from *M*_baseline_ = 40.50 words versus *M*_posttreatment_ = 39.58 words [*z* = −2.874, *p* = 0.004], and Trails A completion time that increased from *M*_baseline_ = 60.27 second versus *M*_posttreatment_ = 60.88 seconds [*z* = −2.117, *p* = 0.034]. These findings although marginally different in some cases produced statistically significant changes over time, albeit mostly with a negative direction. These results imply that this group did not show improvements over time; on the contrary, there were trends of a possible further cognitive decline in the 10-week period between baseline and posttreatment assessments (see Table 5).

### 3.5. Comparison of Composite Cognitive Domain Performance in the RehaCom Group at Baseline, Posttreatment, and Follow-Up

**Figure 2:**
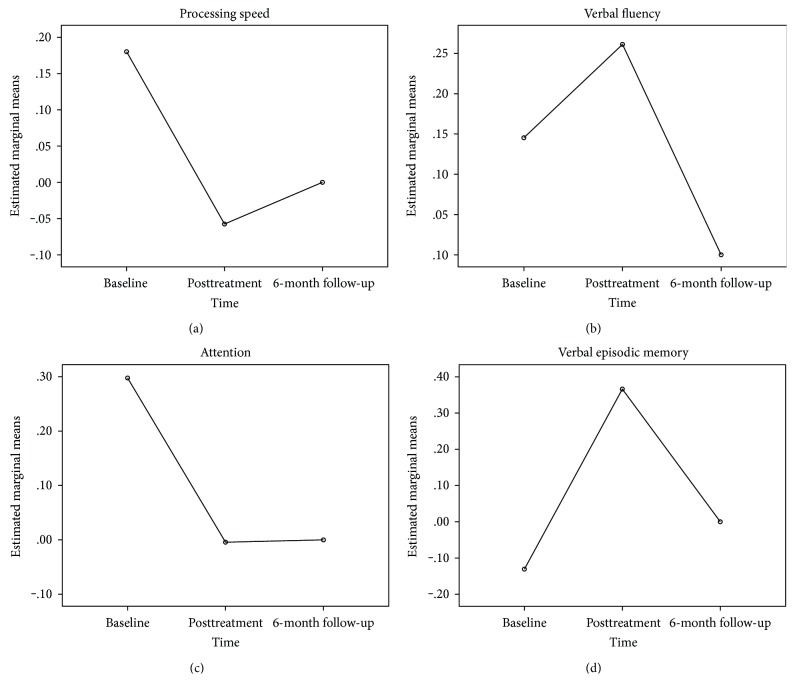
Composite cognitive domain performance (*z*-scores) in the RehaCom group at baseline, posttreatment, and follow-up.

As is evident from Figure 2, we noted a significant reduction (*p* = 0.046) in processing speed from baseline to posttreatment assessment and a slight nonsignificant increase (*p* = 0.067) from posttreatment to follow-up but without dropping to baseline levels of processing speed capacity. Verbal fluency output on the other hand improved significantly from baseline to posttreatment (*p* = 0.034) but showed significant deterioration from posttreatment to follow-up (*p* = 0.020). Attention which was a composite of timed scored measures showed a significant reduction in completion time from baseline to posttreatment (*p* = 0.018) and remained relatively stable over time from posttreatment to follow-up (*p* = 0.290). Verbal episodic memory which reflects total word learning capacity and delayed recall of words showed a significant increase (*p* = 0.002) from baseline to posttreatment and a nonsignificant decrease (*p* = 0.702) from posttreatment to follow-up, but without dropping to baseline levels.

### 3.6. Comparison of Composite Cognitive Domain Performance between the RehaCom Intervention and Control Group at Baseline and Posttreatment

**Figure 3:**
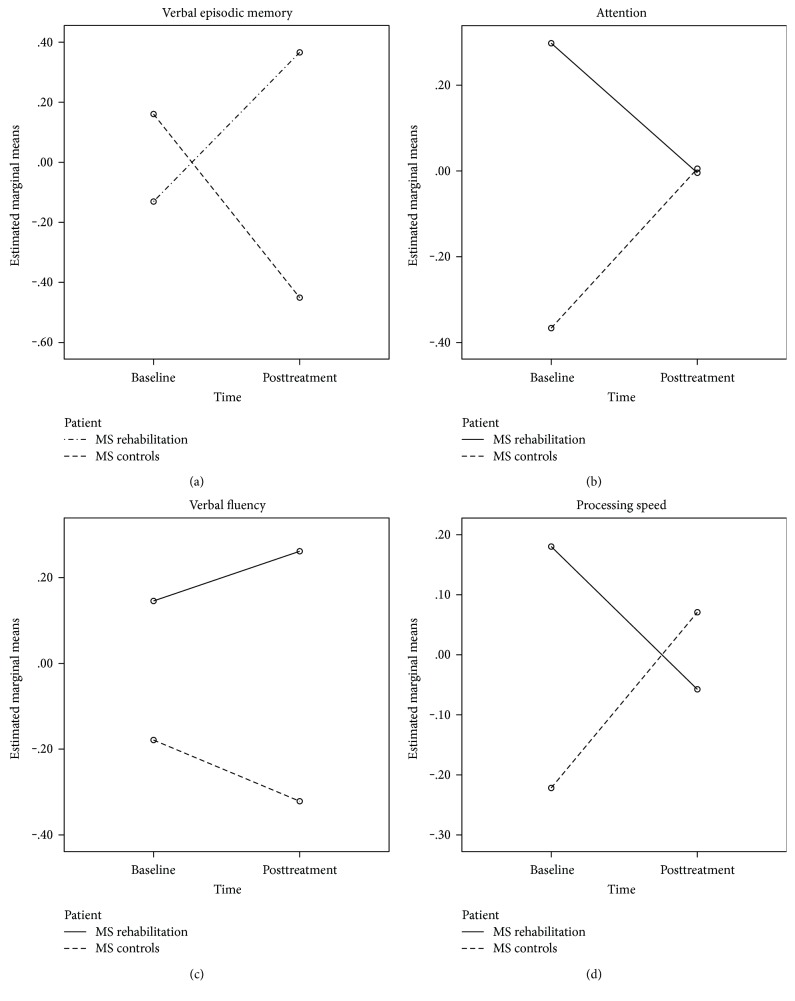
Composite cognitive domain performance (*z*-scores) in the RehaCom intervention and control group at baseline and posttreatment.

As is evident from Figure 3, we found significant composite domain performance differences favoring the intervention group, as an interaction of patient group by time. Specifically, on the verbal episodic memory domain, the rehabilitation group demonstrated a significant increase in the estimated marginal mean over time, indicative of improved encoding, consolidation, acquisition, and delayed recall of verbally learned material, relative to the control group that demonstrated a significant reduction in this domain over the ten-week period. On the attention domain, a composite of timed scored measures and a significant reduction in the estimated marginal mean completion time from baseline to posttreatment were noted, relative to the control group's performance which showed an increase in the estimated marginal mean completion time from pre- to post assessment. A significant reduction was also noted in the estimated marginal mean mental processing speed, from pre- to posttreatment assessment for the intervention group, relative to the control group that demonstrated an increased mental processing speed capacity over this time period. The verbal fluency domain, a composite of phonemic and semantic fluency output, improved significantly for the group that received cognitive treatment, over the 10-week period, from baseline to posttreatment, relative to the control group whose combined fluency output decreased over this time period (see Table 6).

### 3.7. Relationships between Disease Parameters, Depression and Fatigue Level, and Neuropsychological Performance in MS Patients at Baseline

We found significant negative weak correlations between neurological disability status (EDSS) and performance on the SRTLTS (*r* = −0.387, *p* = 0.004), BVMT-R (*r* = 0.305, *p* = 0.010), and SNST (*r* = −0.312, *p* = 0.009) and between disease duration and SRTLTS (*r* = −0.286, *p* = 0.012). We further established a negative relatively large correlation between disability status (EDSS) and performance on the SDMT (*r* = −0.622, *p* = 0.011). Depression and fatigue did not correlate significantly with any of the variables. No other significant correlations were noted between any of the variables.

### 3.8. Satisfaction of Participants Who Completed the Cognitive Rehabilitation Intervention

As mentioned previously in Section 2, we informally asked only cognitively treated patients to provide feedback on a Likert-type Scale ranging from 1 (no benefit) to 5 (large benefit), regarding the personal benefit gained from the intervention on four verbal questions at the posttreatment assessment. All participants who completed the cognitive rehabilitation intervention in the study (IG group; *n* = 32) also responded to the four verbal questions; 93.7% (*n* = 30) of the participants reported large personal benefits gained from the cognitive intervention, improvement of their cognitive abilities, and further noted that they would recommend the intervention to another MS patient; 87.50% (*n* = 28) of the intervention completers reported that the rehabilitation provided large benefits in terms of everyday life activities. Only a minor proportion of the treated patients, 12.50% (*n* = 4), noted gaining a moderate benefit in everyday life activities from the intervention. Satisfaction of the intervention was also evident by the fact that all 32 participants who were randomly allocated to receive cognitive rehabilitation completed the 10-week duration intervention.

## 4. Discussion

In the present study, we conducted a 10-week multicenter randomized controlled trial by utilizing therapeutic modules from the RehaCom software in order to restore the most frequent cognitive domains affected in MS patients. Results revealed that the two groups taking part in the study (IG; intervention and CG; control) were well matched at baseline assessment on demographic and clinical characteristics that could negatively impact the outcome measures and made biased the study results. Moreover, patients' medical therapeutic scheme was well balanced between the two groups.

Following treatment, MS patients showed significant improvement on verbal episodic and visuospatial memory, semantic fluency, processing speed/working memory, response inhibition, attention/visuomotor scanning speed, and set-shifting ability. On follow-up assessment, results revealed a significant reduction on semantic verbal fluency and processing speed performance. All other measures remained relatively stable.

When we compared derived-composite cognitive domain scores in the intervention group, our results revealed a significant decrease in processing speed ability from pre- to posttreatment, which was not retained at follow-up, but also did not drop to pretreatment levels of processing speed capacity. Verbal fluency generation improved significantly from before to after treatment, but this gain was not retained to follow-up. The treatment procedure also produced positive changes in the attention domain, with improved performance been evident at posttreatment, and remaining relatively stable over time for six months. Verbal episodic memory delay-recall domain increased after treatment but decreased marginally in the following 6 months without dropping to pretreatment levels. When cognitive domain performance between the RehaCom intervention group and standard treatment control group was compared over time, we noted that the intervention group outperformed the control group on all derived domains from pre- to posttreatment assessment.

Regarding our hypothesis that control participants who receive standard treatment over the 10-week intervention duration will show either further cognitive decline or remain cognitively stable, we found that performance remained relatively stable over this short duration in most measures, possibly implying that the period of the intervention may be inadequate to produce significant cognitive changes in these patients. An exception to this was the score on the semantic fluency task that deteriorated significantly and the time required to complete Trails A, which increased. Moreover, when composite cognitive domain scores were compared from pre- to posttreatment between the two groups, the control group showed reduced performance relevant to the intervention group. These results indicate a possible trend towards further cognitive decline, suggesting that patients on standard available immunomodulatory treatments are possibly not sufficiently protected against ongoing cognitive decline, although other confounding factors such as depression severity or fatigue changes during this period may have also contributed to these findings. However, as our groups were well matched on possible demographic and clinical confounding variables, these findings are less likely to have been biased by such factors.

The positive findings regarding the amelioration of attention, processing speed, and executive function reported in the present study are in concordance with several other studies that have utilized the RehaCom software in cognitively impaired patients with multiple sclerosis. More specifically, Mattioli et al. [40] reported the effectiveness of a 3-month intensive neuropsychological rehabilitation intervention with the assistance of the RehaCom software on attention, information processing, and executive functions. The same group, Mattioli et al. [9] reported that the cognitive benefits experienced by their MS patients after 3 months of intensive neuropsychological rehabilitation with the assistance of the RehaCom software persisted for 9 months after the rehabilitation onset and also generalized to an amelioration of depression and quality of life. In our study, similar long-term benefits of a shorter, however, duration (6 months) were noted in attentional capacity. In addition to their cognitive and behavioral outcome variables, the authors of the previously mentioned studies provide functional MRI data, suggesting that possible neural correlates of the functional cognitive intervention are training-induced activations of the prefrontal and cingulate cortices, brain structures known to be involved in attention and executive functions. The persistence of cognitive gains over the 9-month period made them believe that it may be related to persistent brain plasticity mechanisms [41].

A structural and functional imaging study by Fillipi et al. [42], which evaluated brain changes and neuropsychological functions in RRMS patients after undergoing computer-assisted cognitive rehabilitation of 12-week duration, utilizing the RehaCom software, with an emphasis on attention, information processing, and executive functions, reported similar positive pre- to posttreatment outcomes on the neuropsychological variables to our study. In addition to providing evidence regarding the efficacy of cognitive training with this software on neuropsychological measures, Fillipi et al. [42] also recorded modifications in the activity of the posterior cingulate cortex (PCC) and dorsolateral prefrontal cortex (DLPC) during the Stroop task, as well as modifications of the activity of the anterior cingulum (AC) and PCC at rest, in the RehaCom-treated group. This study showed that functional cognitive training has the potential to modify the activity of trained neuronal system areas in patients with RRMS, and due to its plasticity mechanisms may recruit additional regions to compensate for cognitively demanding tasks.

Other studies utilizing the RehaCom software have placed emphasis on verbal or visual learning and memory with relatively improved pre- to posttreatment performance differences, similar to the findings noted in our study. In one such study [43], utilizing computer-aided (RehaCom modules) of memory and attention, in a randomized, double-blind controlled trial, noted an improvement in 45% of the studied patients receiving treatment, on a word-list generation task. Another study [44], utilizing the RehaCom software, provided computer-assisted cognitive training for 6 weeks (once weekly) and reported significant improvements of autobiographical memory that were associated with increased cerebral activity in posterior cerebral regions. More recently [45], utilizing the RehaCom package and more specifically similar training modules to the ones utilized in the present study (i.e., attention and concentration, divided attention, logical thinking, and verbal memory) found improved cognitive performance after the training on visual and verbal memory and processing speed. These improvements in cognition were associated with increased functional connectivity in the posterior cingulate cortex (PCC) and inferior parietal cortex (IPC) of the default mode network (DMN), implying training-induced adaptive cortical reorganization in the DMN. This network is considered highly relevant for human cognition under physiological conditions and is the most consistent and commonly reported resting-state network in functional MRI studies of functional connectivity (FC) [46].

Most of the studies mentioned previously had no or relatively short follow-up periods ranging from 3 to 9 months after completion of the training intervention. The long-term persistence of a 15-week domain-specific cognitive training intervention with the RehaCom was reported in a recent two-year follow-up study [47]. The authors report that patients treated with specific cognitive modules aimed at ameliorating the related cognitive domains showed significantly less impaired tests both at one and two-year follow-up assessments relative to a specific group (that received generic psychological intervention). These results further strengthen the available evidence regarding the long-term benefits of relatively short duration (15 weeks in this case) domain-specific functional restorative training with the previously mentioned software program.

Other functional neuroimaging studies have revealed changes in brain activation on task-based functional magnetic resonance imaging (fMRI), change in functional connectivity and, for one study, microstructural changes by diffusion tensor imaging after cognitive rehabilitation [48–50]. These results suggest that restorative or functional training could modify brain functioning and improve network efficiency. The characteristics of change in brain activation and connectivity observed after cognitive rehabilitation interventions (homologous region adaptation, local activation expansion, and extraregion recruitment) and the observed association with neuropsychological improvement suggest that adaptive neuroplasticity may occur after restorative training [51].

Regarding the personal benefit gained from the intervention as rated informally on a Likert-type Scale by the treated patients, the majority reported large benefit and were objectively feeling more confident about their cognitive difficulties and everyday functioning ability. Most patients made special reference to their improved concentration and memory capacity and reduced forgetting rate. They generally felt more confident in performing everyday functional tasks and noted appreciable speed ameliorations in performing tasks that require more rapid actions. As the program was very well received from most patients (this is also evident from the fact that there were no dropouts in the treatment group), they said that they would gladly recommend it to other MS patients.

Although our study has several strengths, including its multicenter randomized controlled design, the well-matched baseline clinical, demographic, and cognitive characteristics of the two groups; the strict inclusion criteria; the absence of comorbid conditions that may have biased the study outcome measures; the ecologically valid treatment intervention modules that were utilized from the RehaCom software; and the noninvasive nature of the intervention do have several potential limitations. Firstly, the present study was not blinded.

Secondly, the control group received only standard clinical care, whereas a placebo intervention applied to this group might have restricted the differentiation of the positive cognitive effects caused by the cognitive rehabilitation intervention. Thirdly, depression and fatigue were assessed only at baseline, and we did not ascertain whether our two groups presented a different evolution of these variables over the intervention period. As our study was not blinded and patients receiving the intervention were offered increased attention, clinical care, and individualized contact on a frequent basis, this may have contributed to the treated patients' general well-being and possibly influenced the positive cognitive outcomes in this group. Finally, we did not utilize formal healthy related quality of life or activity of daily living questionnaires as primary outcome measures. However, in order to evaluate the personal benefit of each patient gained from the intervention, we informally asked treated patients to provide feedback regarding the intervention on four verbal questions at the postintervention assessment.

## 5. Conclusions

In this multicenter randomized controlled trial, we implemented a computer-assisted functional training cognitive rehabilitation intervention of 10-week duration (twice weekly), on cognitively impaired RRMS patients, with low disability status. Our data showed that this relatively short period of domain-specific cognitive training (attention, processing speed, executive functions, and episodic memory) can be helpful in ameliorating the trained functions and that effectiveness persisted at 6-month follow-up for the attention domain. For the other trained domains, performance did not deteriorate to pretreatment levels after 6 months, implying a possible protective long-term effect of the intervention in terms of cognitive deterioration rate. The RehaCom software appears to have sufficient flexibility, dynamics, objectivity, and ecological validity, to make a useful contribution to the clinical practice of cognitive rehabilitation in the MS population. Recent explorative functional neuroimaging studies have reported findings suggesting that cognitive rehabilitation interventions, including those that incorporated the RehaCom software, may induce an increase in the brain activation of treated patients. The contribution of these studies, however, in assessing the impact of cognitive rehabilitation in MS warrants further investigation. Well-designed studies, with clearly defined MS patient populations (e.g., the investigation of cognitive rehabilitation efficacy in progressive MS), and utilization of longer duration and frequency of treatment interventions, with longer follow-up periods, are required in order to elucidate the functional correlates of cognitive amelioration in MS individuals and to make further progress in this rapidly advancing field of cognitive rehabilitation in MS.

## Figures and Tables

**Figure 4:**
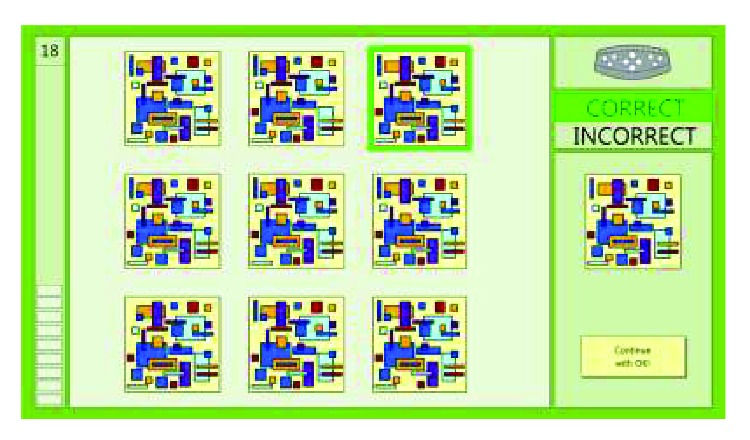
Example of a screen with a 3 by 3 matrix on level 18 of the attention and concentration RehaCom module.

**Figure 5:**
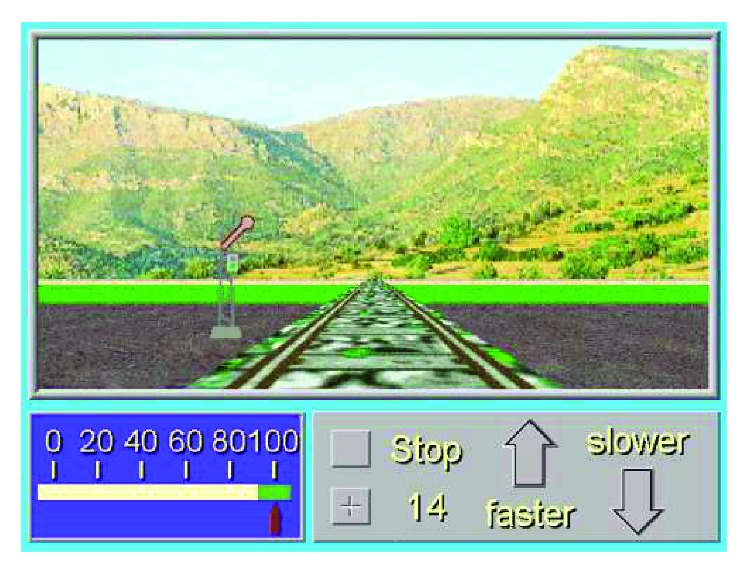
Example of the divided attention task on level 14 of the RehaCom module.

**Figure 6:**
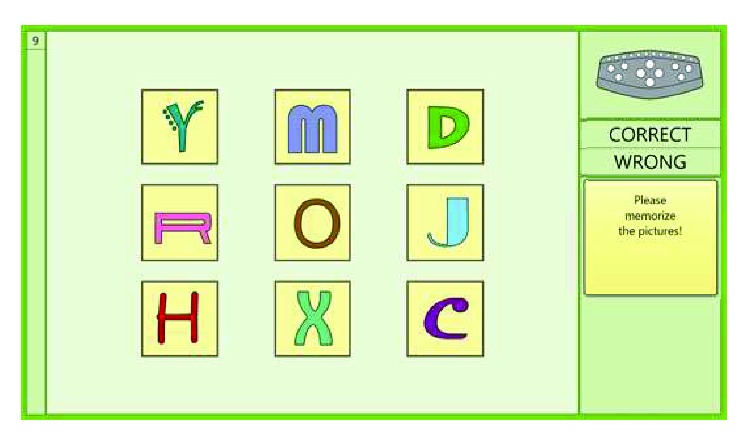
Example training on level 9, of the topological memory task RehaCom module.

**Figure 7:**
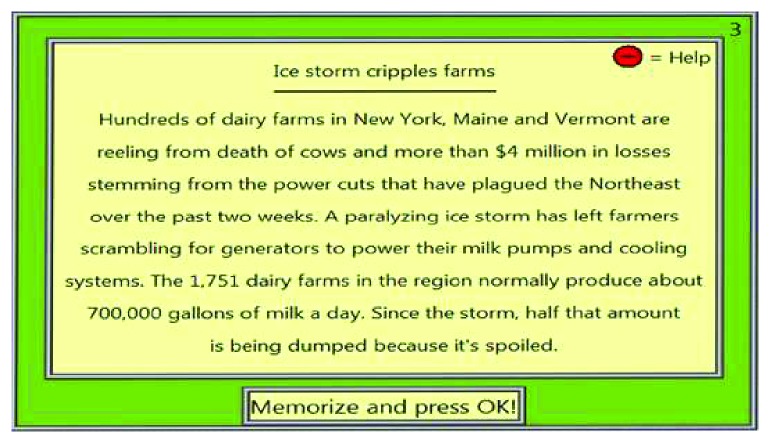
Example of training on level 3, of the verbal memory RehaCom module.

**Figure 8:**
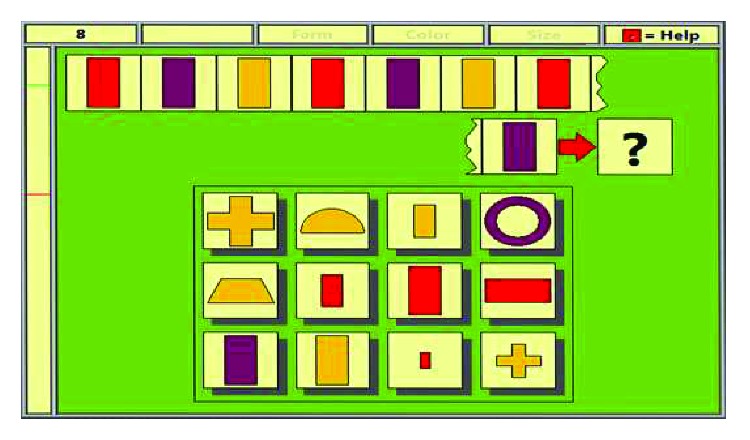
Example of training on level 8, on the logical reasoning RehaCom module.

**Figure 9:**
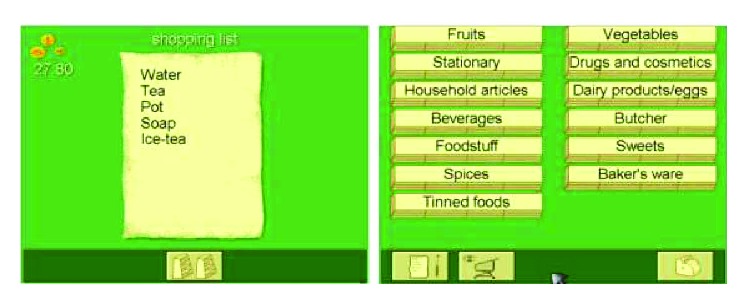
Example of a shopping list on level 14, of the Shopping RehaCom module (from level 11 onward, an amount of money the patient has at his/her disposal is displayed in the upper left of the screen).

**Table 1:** Demographic and clinical characteristics of the sample at baseline.

	MS RehaCom group (*n* = 32) Mean (95% CI) *n* (%)	SD	MS control group (*n* = 26) Mean (95% CI) *n* (%)	SD	*t/U* *x^2^*	df	*p*
Age (years)	46.03 (43.16–48.90)	7.97	45.15 (41.26–49.05)	9.65	0.379	56	0.706
Education (years)	12.12 (10.87–13.38)	3.47	12.73 (11.46–14.01)	3.15	−0.945		0.345
Gender							
Males	10 (31.25)		8 (30.76)				
Females	22 (68.75)		18 (69.24)		0.002	1	0.969
EDSS-median (range)	3.0 (1.5–5.5)		3.5 (1.0–5.0)		−0.126		0.899
Disease duration (years)	13.31 (11.46–15.17)		11.27 (9.39–13.14)		−1.515		0.130
MMSE	27.97 (27.54–28.39)	1.17	28.42 (28.06–28.79)	0.90	−1.578		0.115
WASI (IQ)	102.31 (99.49–105.14)	7.83	103.96 (100.37–107.55)	8.89	−.959		0.338
Premorbid intelligence							
WASI (Voc)			46.2		−0.785		0.680
*T*-score	45.5						
Fatigue (FSS)	4.38 (4.04–4.48)	1.80	4.35 (3.98–4.55)	1.75	−0.297		0.486
BDI-FS	4.31 (3.31–5.32)	2.78	4.46 (3.01–5.91)	3.09	0.178	56	0.859
Medication at enrolment							
Interferon	25 (78.12)		17 (65.38)				
Fingolimod	2 (6.25)		3 (11.53)				
Natalizumab	5 (15.63)		6 (23.07)				

Notes: All values are raw scores. EDSS: Expanded Disability Status Scale; MMSE: Mini Mental State Examination; WASI: Wechsler Abbreviated Scale of Intelligence; WASI (VOC): vocabulary subscale of the Wechsler Abbreviated Scale of Intelligence; FSS: Fatigue Severity Scale; BDI-FS: Beck Depression Inventory-Fast Screen; SD; standard deviation; CI: confidence interval; df: degrees of freedom; *t*: independent sample *t*-test; U: Mann–Whitney *U* test; *x*^2^; chi-squared.

**Table 2:** Comprehensive neuropsychological battery that was administered and arranged by cognitive function/domain assessed.

Cognitive functions/domain assessed	Neuropsychological test used
Verbal memory	Selective Reminding Test (SRT)
Visuospatial memory	Brief Visuospatial Memory Test-Revised (BVMT-R)
Verbal fluency/expressive language	Greek Verbal Fluency Test (phonemic and semantic fluency)
Attention/processing speed	Symbol Digit Modalities Test (SDMT) Trail Making Test Part A
Executive functions	Response inhibition Stroop Neuropsychological Screening Test (SNST)-(colour word task) Set-shifting Trail Making Test Part B (TMT-B)

Note: All measures utilized in the study have been adapted for native Greek speaking adults and demographically corrected normative data have been published (with the exception of the BVMT-R that has been adapted in Greece but normative data are not yet available). The BVMT-R and SDMT have been validated in Greek MS patients. SRT: Selective Reminding Test [24] normative study; BVMT-R: Brief Visuospatial Memory Test-Revised [25] validated in the Greek BICAMS study; SDMT: Symbol Digit Modalities Test [26] normative study and [25] validated in the Greek BICAMS study; Greek Verbal Fluency Test (phonemic and semantic fluency) [27] normative study; SNST: Stroop Neuropsychological Screening Test [28] normative study; TMT-A and TMT-B: Trail Making Test Parts A and B [29, 30] normative studies.

**Table 3:** Performance on neuropsychological measures for the RehaCom and control group at baseline, posttreatment, and at 6-month follow-up.

		MS RehaCom group (*n* = 32) Mean (95% CI)	SD	MS control group (*n* = 26) Mean (95% CI)	SD
SRTLTS	T0	36.72 (34.57–38.86)	5.94	36.42 (34.37–38.48)	5.08
	T1	43.47 (40.55–46.39)	8.09	36.38 (34.34–38.43)	5.06
	T2	43.00 (40.04–45.96)	8.21	—	—

SRTDR	T0	6.09 (5.44–6.75)	1.82	7.15 (6.65–7.66)	1.25
	T1	8.22 (7.59–8.85)	1.75	7.12 (6.73–7.50)	7.12
	T2	7.75 (7.11–8.39)	1.77	—	—

BVMT-RT	T0	21.40 (17.10–24.30)	5.85	22.50 (17.80–25.20)	7.80
	T1	24.50 (19.50–26.30)	6.02	20.80 (17.50–24.60)	6.85
	T2	23.10 (18.90–25.20)	6.40	—	—

VFT phon	T0	31.88 (28.92–34.83)	8.20	29.81 (23.39–30.23)	8.46
	T1	33.13 (30.60–35.65)	7.01	29.95 (24.16–30.53)	7.88
	T2	31.47 (29.20–33.74)	6.29	—	—

VFT sem	T0	41.03 (38.09–43.97)	8.16	40.50 (36.69–44.31)	9.44
	T1	43.56 (40.55–46.57)	8.34	39.58 (35.60–43.55)	9.83
	T2	42.06 (39.05–45.08)	8.35	—	—

SDMT	T0	36.91 (33.89–39.92)	8.36	37.42 (33.03–41.82)	10.87
	T1	40.03 (37.48–42.58)	7.08	37.43 (33.44–41.40)	9.85
	T2	37.50 (35.25–39.75)	6.25	—	—

TMT-A	T0	73.50 (65.08–81.92)	23.35	69.27 (52.05–68.48)	20.30
	T1	59.53 (52.86–66.20)	18.49	68.88 (52.67–69.10)	20.32
	T2	60.31 (53.28–67.34)	19.49	—	—

TMT-B	T0	145. 81 (129.12–162.50)	46.29	111. 54 (96.23–126.84)	37.89
	T1	113.28 (94.72–131.84)	51.47	110.96 (96.18–125.75)	36.60
	T2	115.78 (97.40–134.16)	50.98	—	—

SNST	T0	59.80 (53.30–64.50)	15.50	58.70 (52.60–63.80)	17.30
	T1	63.50 (57.40–68.10)	13.25	57.60 (52.90–62.70)	14.20
	T2	62.10 (56.90–66.20)	14.20	—	—

Notes: All values are raw scores. T0: baseline assessment; T1: posttreatment assessment; T2: 6-month follow-up assessment. MS control group was not assessed at 6-month follow-up. SRTLTS: Selective Reminding Test Long-Term Storage; SRTDR: Selective Reminding Test-Delayed Recall; BVMT-RT: Brief Visuospatial Memory Test-Revised Total Recall; VFT phon: Greek Verbal Fluency Test-Phonemic Fluency; VFT sem: Greek Verbal Fluency Test-Semantic Fluency; SDMT: Symbol Digit Modalities Test; TMT-A and TMT-B: Greek Trail Making Test Part A, Greek Trail Making Test Part B; SNST: Stroop Neuropsychological Screening Test.

**Table 4:** Comparison of neuropsychological test scores for the RehaCom MS-treated group at baseline, posttreatment, and at 6-month follow-up.

	Baseline		Posttreatment		6-month follow-up		Baseline versus posttreatment *p* values	Effect size (*r*)	Baseline versus follow-up *p* values
	Mean	Median	Mean	Median	Mean	Median			
SRTLTS	36.72	36.50	43.47	41.00	43.00	41.00	0.000^∗∗∗^	0.539	0.000^∗∗∗^
SRTDR	6.09	6.50	8.22	8.00	7.75	7.00	0.000^∗∗∗^	0.481	0.001^∗∗^
BVMT-RT	21.40	21.00	24.50	23.90	23.10	23.00	0.000^∗∗∗^	0.469	0.001^∗∗^
VFT phon	31.88	32.00	33.13	33.50	31.47	31.50	ns	—	ns
VFT sem	41.03	40.00	43.56	42.00	42.06	40.50	0.003^∗∗^	0.417	0.424
SDMT	36.91	36.00	40.03	39.00	37.50	37.00	0.000^∗∗∗^	0.522	0.222
TMT-A	73.50	70.00	59.53	62.50	60.31	66.00	0.000^∗∗∗^	0.573	0.000^∗∗∗^
TMT-B	145.81	32.50	113.28	106.50	115.78	107.50	0.000^∗∗∗^	0.506	0.000^∗∗∗^
SNST	59.80	58.50	63.50	62.90	62.10	60.40	0.000^∗∗∗^	0.460	0.000^∗∗∗^

Notes: All values are raw scores (^∗∗∗^*p* < .001 and ^∗∗^*p* < .01). Friedman's nonparametric test used for comparison of medians between baseline, posttreatment, and follow-up. Wilcoxon test with Holm-Bonferroni correction used for pairwise comparisons. Effect size (*r*) for Wilcoxon test calculated as follows: *r* = *z*/√*N*(*N* = total number of samples)*;* abs (*r*) 0.1 small size; 0.3 medium size; 0.5 large size; ns: Friedman's test indicated no significant group effect for VFT phon. SRTLTS: Selective Reminding Test Long-Term Storage; SRTDR: Selective Reminding Test-Delayed Recall; BVMT-RT: Brief Visuospatial Memory Test-Revised Total Recall; VFT phon: Greek Verbal Fluency Test-Phonemic Fluency; VFT sem: Greek Verbal Fluency Test-Semantic Fluency; SDMT: Symbol Digit Modalities Test; TMT-A and TMT-B: Greek Trail Making Test Part A and Greek Trail Making Test Part B; SNST: Stroop Neuropsychological Screening Test.

**Table 5:** Comparison of neuropsychological test scores for the standard care MS control group at baseline and posttreatment.

	Baseline		Posttreatment		Baseline versus posttreatment *z-*score	*p* values	Effect size
	Mean	Median	Mean	Median			
SRTLTS	36.42	36.50	36.38	37.00	0.187	0.852	0.026
SRTDR	7.15	7.00	7.12	7.00	0.302	0.763	0.042
BVMT-RT	22.50	22.00	20.80	21.10	0.304	0.675	0.034
VFT phon	29.81	28.00	29.95	28.50	−2.365	0.018^∗^	0.328
VFT sem	40.50	39.50	39.58	38.50	−2.874	0.004^∗∗^	0.399
SDMT	37.42	38.50	37.43	39.00	−0.069	0.945	0.010
TMT-A	60.27	57.00	60.88	58.50	−2.117	0.034^∗^	0.294
TMT-B	111.54	110.00	110.96	107.50	1.042	0.298	0.144
SNST	58.70	57.40	59.10	57.60	0.348	0.780	0.035

Notes: All values are raw scores. ^∗∗^*p* < 0.01 and ^∗^*p* < 0.05. Wilcoxon signed-ranked nonparametric test used for comparison of medians between baseline and posttreatment. SRTLTS: Selective Reminding Test Long-Term Storage; SRTDR: Selective Reminding Test-Delayed Recall; BVMT-RT: Brief Visuospatial Memory Test-Revised Total Recall; VFT phon: Greek Verbal Fluency Test-Phonemic Fluency; VFT sem: Greek Verbal Fluency Test-Semantic Fluency; SDMT: Symbol Digit Modalities Test; TMT-A and TMT-B: Greek Trail Making Test Part A and Greek Trail Making Test Part B; SNST: Stroop Neuropsychological Screening Test.

**Table 6:** Two-way mixed effect ANOVA for cognitive domain performance: time (within subjects' factor) and patient group: (between subjects' factor).

	Verbal episodic memory	Attention	Verbal Fluency	Processing Speed
Time	0.628	0.727	0.767	0.662
Group	0.171	0.099	0.047	0.522
Time × group	<0.001	<0.001	0.006	<0.001

## Appendix

### Detailed Description of the RehaCom Modules Used in the Cognitive Rehabilitation Intervention

As noted previously, in this specific study, as most of our MS patients that took part in the intervention were impaired in more than one cognitive domain but mostly on episodic memory, information processing speed/attention and executive functions, the intervention was balanced over the 10-week period in order to train all domains equally.

In order to train attention, we used two modules. The first module is called *attention and concentration*, training mainly selective attention, and in this procedure, a separately presented picture is compared to a matrix of pictures. The patient has to recognize a picture (symbols, items, animals, or abstract figures) and respond by selecting it from a matrix. This activity trains the ability to differentiate and concentrate simultaneously. The matrices are either 3 pictures (1 × 3 matrix), 6 pictures (2 × 3 matrix), or 9 pictures (3 × 3 matrix) depending on the level of difficulty that we want to train (see Figure 4).

The second module used to train attention is a more naturalistic or ecologically valid cognitive task called *divided attention.* In this task, the patient works through the cognitive training as the driver of a train shown on the lower part of the screen. He sits in the steeple cab (or driver's cab) of the train and can observe the railway like looking through the windscreen of the driver's cab and has the following task: he must carefully observe the control panel of the train and the countryside, as it flashes past, and react to different events as they occur. At first, only the acceleration of the train is to be regulated. Later, and with increasing levels of difficulty, more tasks are added, in which different levels of attention and particular reactions are expected from the trainee. The driver's panel contains a speedometer, a so-called “Deadman's lamp” and the “emergency stop lamp.” On the speedometer, a “target speed” is set that the patient must retain. As soon as a lamp lights up, the patient has to press the corresponding button on the RehaCom Panel (e.g., the stop button). If a relevant object appears on the railway, the patient also has to react to it (e.g., stopping at a red signal) (see Figure 5).

In order to train memory, we also used two different modules. For training visuospatial memory, we used the *topological memory* module. In the so-called “memorizing phase,” a variable number of cards (depending on the level of difficulty) with concrete pictures or geometric figures are displayed on the screen. The patient has to memorize the position of the pictures. After a preset time—or manually by pressing the OK button—the pictures of the matrix are hidden (turned face down). The patient must find the picture matching the one indicated on the right side of the screen. Altogether, 464 pictures of concrete objects, geometric figures, and letters are available. The number of simultaneously displayed cards varies from 3 to a maximum of 16 (see Figure 6).

In order to train verbal episodic memory, we used the *verbal memory* module. In this task, a short story is presented on the screen. The patient has to memorize as many details of the story as possible (names, numbers, events, and objects). The learning phase is completed by pressing the OK button. After that, the patient must answer questions about the content of the story. More than 80 short stories are available. Depending on the setting, either the computer or the therapist selects a story for the patient (see Figure 7).

Executive functions were trained with two respective modules. The first module called *logical reasoning* trains abstract logical thinking ability and conclusive thinking. The task requires that from several symbols (pool of answers), the client has to find out the one that correctly continues a given sequence of symbols. A sequence of symbols (circles, triangles, squares, etc.) of different shape, color, and size is displayed on the screen being in a regular relation to each other. If the answer is wrong, special pieces of information about the type of error (shape, color, and/or size) are provided. The principle behind the training is that the problem-solving tasks are graphic and vivid. The patient learns to recognize the concepts underlying each problematic situation and uses these concepts to find a solution to the logic problem (see Figure 8).

The second module used to treat executive function is a more ecologically valid highly realistic training exercise called *shopping*. The patient performs the same tasks on the computer that he would have to do while going shopping in a supermarket. Specifically, the trainee gets a shopping list of articles that he has to look for in a supermarket and put into a trolley. When all articles are in the trolley, the trainee can leave the supermarket by using the “cash” button. Beyond a certain level of difficulty, additional demands are made on the trainees' mathematical abilities (a certain amount of money is specified, the products are marked with prices, etc.). This training module currently uses more than 100 articles illustrated photorealistically (food, household objects, etc.). These articles appear on shelves from which the client must choose them. The training programme disposes of a voice output, which means all articles are named when selected (see Figure 9).
